# Cottonseed oil alleviates ischemic stroke injury by inhibiting ferroptosis

**DOI:** 10.1002/brb3.3179

**Published:** 2023-07-21

**Authors:** Miao Sun, Min Liu, Qingxiao Li, Xiaoying Zhang, Siyuan Liu, Huikai Yang, Le Yang, Jiahe Tian, Weidong Mi, Yulong Ma

**Affiliations:** ^1^ Department of Anesthesiology The First Medical Center of Chinese PLA General Hospital Beijing China; ^2^ Department of Anesthesiology The First Affiliated Hospital Jinzhou Medical University Jinzhou Liaoning Province China; ^3^ Department of Anesthesiology Beijing Tongren Hospital, Capital Medical University Beijing China; ^4^ Department of Nuclear Medicine The First Medical Center of Chinese PLA General Hospital Beijing China; ^5^ Department of Anesthesiology Affiliated Hospital of Nantong University Nantong Jiangsu Province China; ^6^ Department of Pharmacy Tangdu Hospital Air Force Military Medical University Xi'an Shaanxi Province China

**Keywords:** ^68^Ga‐citrate, cottonseed oil (CSO), ferroptosis, ischemic stroke, neuroprotection, positron emission tomography (PET)

## Abstract

**Introduction:**

Ferroptosis has recently been recognized as a new cause of ischemia reperfusion injury due to blood–brain barrier (BBB) disruption followed by secondary iron‐loaded transferrin (TF) influx. As a novel and independent cell death pathway, ferroptosis was characterized by iron‐dependent lipid peroxidation, decline of GSH, GPX4, and shrinking mitochondria. Cottonseed oil (CSO), a liposoluble solvent, can alleviate ischemia stroke injuries and oxidative stress. However, the effect of CSO on ischemic stroke–induced ferroptosis has not been explored. In this study, we investigated the effect of CSO on ferroptosis caused by cerebral ischemic injury in rats.

**Methods:**

We conducted the subcutaneous injection of 1.3 mL/kg CSO every other day for 3 weeks on rats with middle cerebral artery occlusion–reperfusion (MCAO‐R) injury. We used Garcia Test, TTC staining, HE, Nissl and NeuN staining, Evans blue test, ^68^Ga‐citrate PET, Western blot, immunofluorescence staining, Elisa kits, and transmission electron microscopy to detect the infarct volume, neural injuries, and ferroptosis‐related indexes.

**Results:**

CSO treatment could significantly ameliorate MCAO‐R‐induced neurological dysfunction in a male rat model. Furthermore, it reduced infarct volume and neuronal injuries; protected BBB integrity; reduced the influx of iron ion, TF, and TF receptors; up‐regulated anti‐ferroptosis proteins (GPX4, xCT, HO1, FTH1), while down‐regulating ferroptosis‐related protein ACSL4; increased the activity of GSH and SOD; and decreased MDA and LPO levels. Mitochondrial destruction induced by ischemic stroke was also alleviated by CSO treatment.

**Conclusion:**

CSO treatment can alleviate ischemic stroke injury via ferroptosis inhibition, which provides a new potential therapeutic mechanism for CSO neuroprotection against ischemic stroke.

## Graphical abstract

CSO treatment alleviates ischemic stroke induced ferroptosis. 68Ga‐citrate can be used to evaluate the iron levels after stroke. CSO treatment regulates the ferroptosis related proteins.

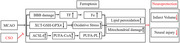

## 1 INTRODUCTION

Stroke is one of the leading causes of death and disability‐adjusted life‐years worldwide (Campbell et al., 2019). Management of intravenous thrombolysis and endovascular thrombectomy within 4–6 h of stroke onset can benefit patients (Sandercock et al., 2012). Still, few patients successfully receive effective cure because of the relatively narrow time window (Yeo et al., 2013), and despite extensive efforts, clinical trials for acute stroke treatment drugs ended in failure (Phipps & Cronin, 2020). Therefore, novel stroke therapy should focus on daily prevention.

Cottonseed oil (CSO) is a vegetable oil, commonly used to dissolve lipid soluble drugs (Aizawa et al., 2011). CSO has been previously reported to protect against intestinal inflammation, tumor metastasis, oxidative stress, and atherosclerosis (Araujo et al., 2019; Mahley et al., 1976; Miura et al., 2007; Park et al., 2019), but little attention has been paid to the effect of CSO on central nervous system. We previously reported that CSO pretreatment can significantly ameliorate ischemic stroke injury by inhibiting glial neuroinflammation and oxidative stress (Liu et al., 2020; Liu et al., 2021). High purity CSO is an edible oil; hence, it may become a new pretreatment strategy to prevent stroke. However, the underlying mechanisms of CSO's neuroprotective effects against ischemic stroke remain largely unknown.

Ferroptosis is a new pathogenic cell death pathway characterized by iron‐dependent lipid peroxidation, decrease of glutathione (GSH), glutathione peroxidase 4 (GPX4), and abnormal mitochondrial morphology (Dixon et al., 2012). Recent studies found that ferroptosis plays an important role in cerebral ischemic injury, with different pathological features and processes such as neuroinflammation, neural excitotoxicity, oxidative stress, and apoptosis (Shen et al., 2020). Anti‐ferroptosis agents such as ferrostatin‐1 and liproxstatin‐1 can alleviate neural damage in rodent middle cerebral artery occlusion (MCAO) models (Chen et al., 2021; Zhu et al., 2022). Therefore, ferroptosis has been regarded as a new target to reduce ischemic stroke injuries, but the effect of CSO on ferroptosis induced by ischemic stroke has not been explored so far.

In this study, we explored the effect of CSO on ferroptosis induced by ischemic stroke and its underlying mechanism. Treatment with CSO significantly attenuated the ischemic stroke injury and blood–brain barrier (BBB) disruption; reduced iron accumulation, lipid peroxidation, and mitochondrial destruction; and regulated the key proteins of ferroptosis including GPX4, long‐chain acyl‐CoA synthetase 4 (ACSL4), transferrin (TF), TF receptor, SLC7A11 (xCT), heme oxygenase 1 (HO1), and ferritin heavy chin 1 (FTH1).

## 2 METHODS AND MATERIALS

### 2.1 Animals

Male Sprague‐Dawley (SD) rats, aged 8–10 weeks and weighing between 280 and 350 g, were procured from the Laboratory Animal Center of Chinese PLA General Hospital with a specific pathogen‐free grade. The rats were housed in an environment with controlled temperature (22 ± 3°C) and humidity (50%−70%), under a 12‐h light/dark cycle, with ad libitum access to food and water. The Ethics Committee for Animal Experimentation of the Chinese PLA General Hospital approved all experimental procedures.

A total of 240 rats were randomized into four groups. (A) Sham group: sham plus phosphate‐buffered saline (PBS), *n* = 50; (B) Sham+CSO group: sham plus CSO treatment group, *n* = 50; (C) MCAO group: middle cerebral artery occlusion–reperfusion (MCAO‐R) injury plus PBS, *n* = 70; (D) MCAO+CSO group: MCAO‐R injury plus CSO treatment, *n* = 70.

### 2.2 CSO treatment

The rats in Sham‐CSO and MCAO‐CSO groups received subcutaneous injection of 1.3 mL/kg CSO (J&K, China) every alternate day for a duration of 3 weeks as previously described (Mahley et al., 1976). In contrast, rats in Sham and MCAO groups were injected with an equivalent volume of PBS. Researchers were blinded to experimental grouping.

### 2.3 Middle cerebral artery occlusion and reperfusion (MCAO‐R)

The MCAO‐R model was established on rats in MCAO and MCAO+CSO groups by researchers blinded to experimental grouping as previously described (Liu et al., 2020). Briefly, after general anesthesia with 3% pentobarbital sodium (30 mg/kg), rats were put on thermostatic blankets to maintain body temperature (36.5−37.5°C) under continuous monitoring by thermometers. Following exposure of the right common carotid artery (CCA) from a midline skin incision, a suture (RWD, USA) was inserted from the left CCA to the origin of the MCA to block blood flow for 1 h. Regional cerebral blood flow was monitored at time point of pre‐ischemia, during ischemia and reperfusion by Laser speckle flowmetry (RWD Life Science Co, USA) according to manufacturer's instructions. The physiological indices, including mean arterial blood pressure (MABP), temperature, glucose, hematocrit, pH, pO2, and pCO2 were examined. No difference was observed between the four groups (Data S1). Rats with a mean cortical cerebral blood flow decrease to 30% of the pre‐ischemic level and which recovered to 70% of the baseline after reperfusion were selected for subsequent experiments. In the MCAO group, 18 rats were dead and 2 rats were excluded; and in the MCAO+CSO group, 15 rats were dead and 5 rats were excluded.

### 2.4 Garcia test

The Garcia test was performed by a blinded observer and confirmed by another observer to evaluate neurological deficits after 24 h of reperfusion (*n* = 10) (Bachour et al., 2016). Garcia test included six factors: (1) spontaneous activity (0–3 points), (2) symmetry of limb movement (0–3 points), (3) forepaw outstretching (0–3 points), (4) climbing cage (1–3 points), (5) body proprioception (1–3 points), and (6) vibrissae reaction (1–3 points). The total score ranged from 3 points (severe impairment) to 18 points (no impairment) according to the neurological function after ischemia injury.

### 2.5 Measurement of brain infarct volume

To determine the infarct volume in vitro, 2,3,5‐triphenyltetrazolium chloride (TTC) staining was used to quantitatively assess the infarct volume as previously described (*n* = 10) (Liu et al., 2020). Six serial brain section slices were photographed and the infarct size (white, unstained area) of both TTC staining and MRI was analyzed with Image J (National Institutes of Health, USA) by operator blinded to groups. The infarct volume was calculated to assess edema recovery. The total relative infarct size = sum of relative infarct volume (contralateral area−ipsilateral non‐infarct area) from 6 sections/sum of contralateral area from 6 sections.

### 2.6 HE staining and Nissl staining

After general anesthesia as described above, the rats went through transcardial perfusion with 0.9% pre‐cooled saline and 4% paraformaldehyde to fix their brains. Brains were then embedded in paraffin and sectioned into 4‐μm slices for hematoxylin and eosin (HE) staining and Nissl staining. Sections (*n* = 3) were stained with HE (Wuhan Servicebio Technology Co. Ltd., China) to detect the pathological changes in ischemic penumbra 24 h after reperfusion. Nissl staining of the sections (*n* = 3) was performed according to the manufacturer's manual of Nissl staining kit (Solarbio, China) to detect neuronal morphologic changes in ischemic penumbra 24 h after reperfusion. Observers were blinded to groups and counted the numbers of HE‐positive cells and Nissl‐positive neurons in the penumbra, respectively, in five different fields of view for each section by light microscopy (Olympus, Japan).

### 2.7 Immunofluorescence staining

Immunofluorescence staining was conducted as described in our previous studies (Liu et al., 2020; Liu et al., 2021). The frozen coronal sections of rat brains in the ischemia penumbra were achieved 24 h after MCAO‐R (*n* = 3) for NeuN staining and GPX4 staining. The following antibodies were used: mouse anti‐NeuN (Abcam, ab252833, 1:100), mouse anti‐GPX4 (Santa Cruz, sc‐166570, 1:50), and Alexa‐488 (Abcam, ab150105, 1:200) donkey anti‐mouse secondary antibody. The total numbers of NeuN‐positive neurons and GPX4‐positive cells in the ischemic penumbra were counted in five different fields of view for each section by a researcher who was blinded to the groupings.

### 2.8 Assessment of the blood–brain barrier permeability

The BBB permeability was measured by Evans blue method. A total of 4 mL/kg of 2% Evans blue (Sigma Aldrich, Germany) was injected into the right jugular vein (*n* = 6) after removing the suture. Rats were humanly euthanized 24 h after reperfusion; the brains sectioned into 2 mm slices and photographed with a digital camera.

The ischemic hemispheres (*n* = 6) were homogenized in N, N‐dimethylformamide (10 mL/kg, Sigma Aldrich), incubated for 18 h at 55°C, and then centrifuged at 3000 rpm for 10 min. The EB content in the supernatants was calculated based on standard curve by spectrophotometry at 620 nm to quantitatively measure BBB permeability change.

### 2.9 Brain edema assessment

Brain water content was measured to investigate brain edema. Twenty‐four hours after MCAO‐R, rats (*n* = 6) were anesthetized and the brains collected on ice. A coronal brain section was obtained by cutting off tissues between 3 and 8 mm from the frontal pole. Brain slices were then separated into ischemic and non‐ischemic hemispheres. Fresh ischemic hemisphere samples were weighed and then dried at 100°C in a vacuum oven for 48 h and reweighed. Brain water content (%) was calculated as ((wet weight − dry weight)/wet weight) × 100%.

### 2.10 Micro‐positron emission tomography (micro‐PET) and biodistribution

Positron emission tomography (PET) is widely used in clinical trials and animal research for diagnosis and drug development as it provides a noninvasive pathway to investigate some specific molecular changes in the brain (Yamasaki et al., 2021). TF is a key protein to induce ferroptosis during ischemic injuries, and it is hard to pass through the BBB in normal condition. Therefore, we used ^68^Ga‐citrate which can bind to pro‐transferrin to probe for PET imaging and evaluate iron levels and BBB integrity. Biodistribution was applied for quantitative analysis of the ^68^Ga‐citrate levels in brain tissues.

^68^Ga‐citrate was synthesized by the Department of Nuclear Medicine, the First Medical Center of Chinese PLA General Hospital, as previously described (Behr et al., 2018), and its radiochemical purity was over 99%. Twenty‐four hours after reperfusion, 200 μCi of ^68^Ga‐citrate of 0.1 mL total volume was injected via the tail vein, and micro‐PET scanning was performed after 1.5 h. PET scanning was completed by the SIGNA PET/MR scanner (Antpedia, China) with the induction of anesthesia by isoflurane (RWD Life Science Co, USA). Rats were in the prone position and got 30 min PET scan and 10 min CT scan. The images of CT, PET, and fusion of the rat brain were captured on the Recon/Avator‐s‐10 workstation. The max standardized uptake value (SUVmax) of region of interest (ROI) was measured and calculated (*n* = 3). The ROI was selected as the ischemic hemisphere of three representative coronal pictures of brains. A radiologist blinded to experimental groups operated the scan examination and analysis.

The γ‐counter (Hidex, German) was used to detect the radioactive value of ^68^Ga‐citrate in ischemic penumbra tissues 24 h after reperfusion, and the results were calculated after time attenuation correction (%ID/g). Briefly, 1.5 h after injection of 15 μCi of ^68^Ga‐citrate in 0.1‐mL solution, rats (*n* = 5) were anesthesia by pentobarbital sodium (1.3 mg/kg), and the ischemic penumbra of rat brain tissues was collected as above. The results were calculated by a radiologist blinded to the experimental groups.

### 2.11 Western blot analysis

The ischemic penumbra of rat brain tissues (*n* = 6) was collected for Western blot analysis as previously described (Liu et al., 2020). The following primary antibodies were used: anti‐beta‐Actin (Abcam, ab8226, 1:1000), anti‐GPX4 (Abcam, ab252833, 1:1000), anti‐FTH1 (Abcam, ab183781, 1:1000), anti‐TF (Abcam, ab82411, 1:1000), anti‐TF receptor (Abcam, ab269513, 1:1000), anti‐HO1 (Abcam, ab68477, 1:1000), anti‐ACSL‐4 (Abcam, ab155282, 1:1000), and anti‐xCT (Invitrogen, PA1‐16893, 1:1000).

### 2.12 Determination of malondialdehyde, glutathione, and superoxide dismutase

The ischemic penumbra of brain tissues was collected 6 h after reperfusion and homogenized in pre‐cooled PBS (*n* = 6). The tissue fluid was used to detect MDA, GSH, SOD with malondialdehyde (MDA) assay kit (Nanjing Jiancheng, China), reduced glutathione (GSH) assay kit (Nanjing Jiancheng, China), and superoxide dismutase (SOD) assay kit (Nanjing Jiancheng, China), respectively. All procedures followed the manufacturer's protocols. The total protein content of tissue fluid was analyzed by the BCA protein detection kit (Beijing Soleibao Biotechnology, China).

### 2.13 Measurement of iron levels and lipid peroxidation

The tissue fluid was collected 24 h after reperfusion as described above for detecting iron and LPO levels by using an iron assay kit (Nanjing Jiancheng, China) and lipid peroxidation assay kit (Nanjing Jiancheng, China) according to the manufacturer's protocols. The total protein content was analyzed by the BCA protein detection kit (Beijing Soleibao Biotechnology, China).

### 2.14 Transmission electron microscopy

Twenty‐four hours after MCAO‐R, rats (*n* = 3) were anesthetized and intracardially perfused with cold phosphate‐buffered solution (PBS) containing 2.5% glutaraldehyde (Servicebio, China). Brain tissues, 1 mm^3^, obtained from the ischemia penumbras of rat brains were prepared for TEM observation as previously described (Li et al., 2021). The changes in the mitochondrial structure under TEM were assessed by an observer blinded to experimental groups.

### 2.15 Statistical analysis

All data were analyzed by an observer blinded to experimental groups. Statistical calculations were performed using GraphPad Prism software (v. 9.0). Data was presented as the mean ± SD and comparisons among multiple groups were performed using one‐way ANOVA followed by Tukey's post hoc test, while neurological deficit scores were presented as median with interquartile range and analyzed using two‐tailed Mann–Whitney U tests. *p*‐Value < .05 was considered statistically significant.

## 3 RESULTS

### 3.1 CSO treatment significantly attenuated cerebral ischemic injury and neuronal injury

**FIGURE 1:**
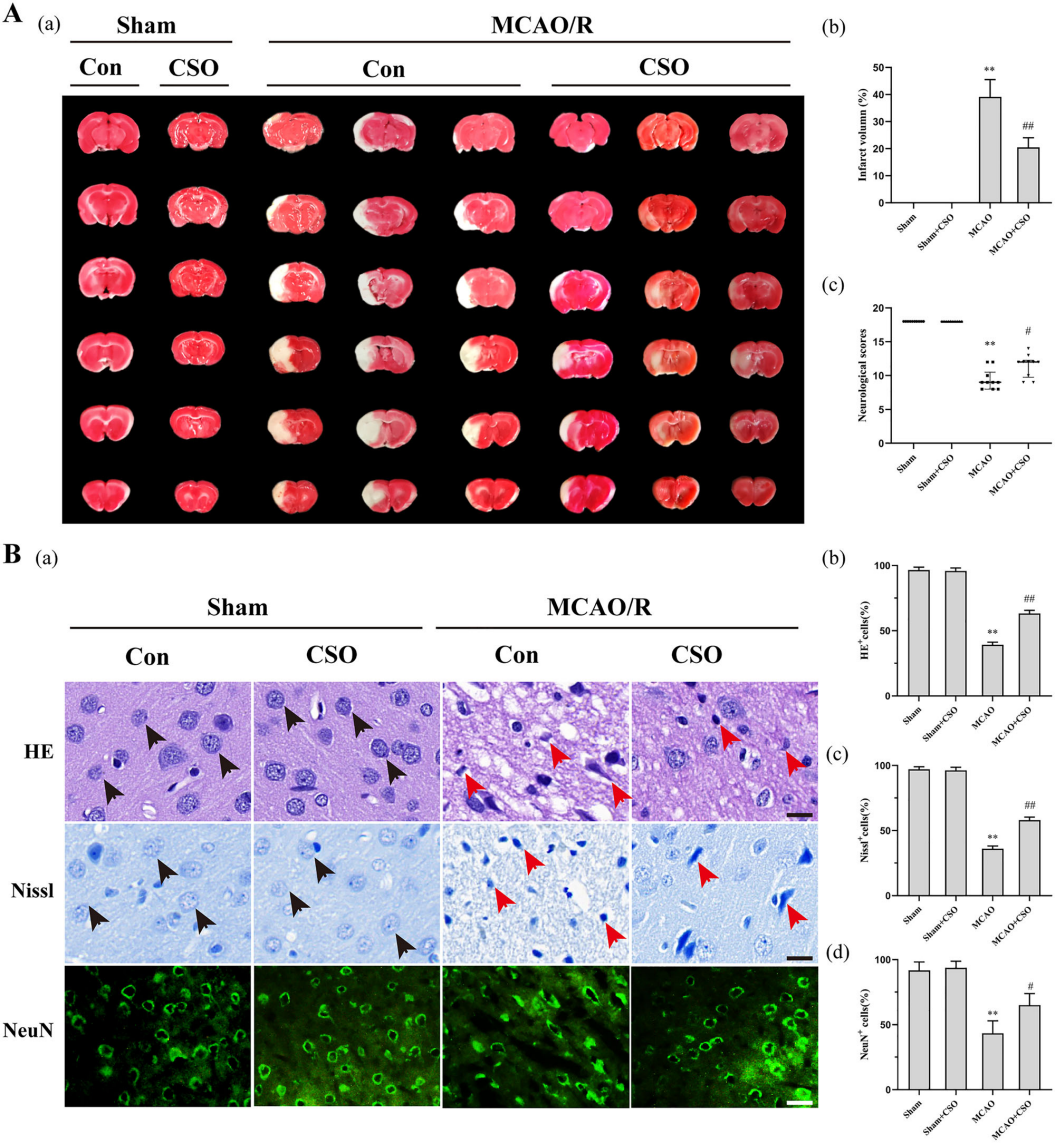
Cottonseed oil (CSO) treatment reduced ischemia stroke injuries. (A‐a) Representative photographs of brain slices reflecting infarct volume 24 h after reperfusion. (A‐b) Statistical analysis of infarction volume in four groups. **p < .01 versus Sham group, ^##^ *p* < .01 versus MCAO group, *n* = 10 per group. (A‐c) Garcia scores tested 24 h after reperfusion. **p < .01 versus Sham group, ^#^ *p* < .05 versus MCAO group, *n* = 10 per group. (B‐a) HE staining showing cell morphology in the ischemic penumbra 24 h after reperfusion. Scale bars = 20 μm. Black arrows indicate normal cells with contact structure and compact arrangement. Red arrows represent abnormal cells with damaged structure and sparse arrangement. Nissl staining showing neuronal morphological changes in the ischemic penumbra 24 h after reperfusion. Scale bars = 20 μm. Black arrows represent intact neurons with flush cell bodies. Red arrows represent injured neurons with shrunken cell bodies and nuclei. NeuN staining showing survival neurons in the ischemic penumbra 24 h after reperfusion. Scale bars = 20 μm. (B‐b) Statistical analysis of HE positive cells in four groups. **p < .01 versus Sham group, ^##^ *p* < .01 versus MCAO group, *n* = 3 per group (b). (B‐c) Statistical analysis of Nissl positive cells in four groups. **p < .01 versus Sham group, ^##^ *p* < .01 versus MCAO group, *n* = 3 per group. (B‐d) Statistical analysis of NeuN positive cells in four groups. **p < .01 versus Sham group, ^#^ *p* < .05 versus MCAO group, *n* = 3 per group.

To assess the cerebral ischemic injuries, infarct volume was measured by TTC staining in the four groups. As shown in Figure 1A(a,b), rats in Sham and Sham+CSO had no infarct area. There was a significant effect for the measured infarct volume across all four groups (*F*(3,20) = 158.1, *p* = .001). Post hoc comparisons using Tukey HSD test suggested that the volumes for the MCAO group (39.1 ± 6.4%) were significantly larger than the Sham (0 ± 0%) and Sham+CSO (0 ± 0%) groups. Rats in the MCAO+CSO group had reduced infarct volume than those in the MCAO group (20.5 ± 3.5%).

Neurological scores were evaluated to measure the neurological function of the four groups (Sham, Sham+CSO, MCAO, MCAO+CSO) after MCAO‐R injury. As shown in Figure 1A‐c, there was no neurological deficit in the Sham and Sham+CSO groups; the scores of the MCAO group were clearly lower than the Sham group, indicating worse neural injuries (*Z* = −4.075, *p =* .001). However, compared with the MCAO group, CSO treatment significantly attenuated the neurological deficit (*Z* = −2.551, *p* = .013).

We used HE, Nissl, and NeuN staining to exhibit the neuronal damage and pathology of ischemic penumbra after the MCAO‐R injury (Figure 1B). As shown in Figure 1B‐a, the HE‐positive cells showed a clear boundary and dense structure with complete nucleolus. There was a significant effect for the proportion of HE‐positive cells across all four groups (*F*(3,8) = 456.3, *p =* .001). Post hoc comparisons using Tukey HSD test suggested that the proportion of HE‐positive cells in the MCAO group (39.1 ± 2.1%) was significantly lower than the Sham (96.5 ± 2.2%) and the Sham+CSO (95.8 ± 2.3%) groups. Rats in the MCAO+CSO group (63.2 ± 2.4%) had increased proportion of HE‐positive cells than those in the MCAO group. As shown in Figure 1B‐a, Nissl‐positive cells showed intact neurons with flush cell bodies while shrunken cell bodies with shrunken and pyknotic nuclei were found in injured neurons. As shown in Figure 1B‐c, there was a significant effect for the proportion of Nissl‐positive cells across all four groups (*F*(3,8) = 556.5, *p* = .001). Post hoc comparisons using Tukey HSD test suggested that the proportion of Nissl‐positive cells in the MCAO group (36.0% ± 2.1%) was significantly lower than the Sham (97.0% ± 2.0%) and the Sham+CSO (96.3% ± 2.3%) groups. Rats in the MCAO+CSO group (58.0 ± 2.4%) had increased proportion of Nissl positive cells than those in the MCAO group. As shown in Figure 1B‐a, the NeuN positive cells in MCAO group were less than those in Sham and MCAO+CSO groups. There was also a significant effect for the proportion of NeuN positive cells across all four groups (*F*(3,8) = 28.65, *p* = .001). According to the post hoc comparisons using Tukey HSD test, the proportion of NeuN positive cells in MCAO group (43.3 ± 9.6%) was significantly lower than the Sham (91.7 ± 6.5%) and Sham+CSO (93.7 ± 5.1%) groups. Rats in the MCAO+CSO group (65.0 ± 8.9%) had more NeuN positive cells than those in the MCAO group.

### 3.2 CSO treatment significantly reduced the stroke‐mediated BBB disruption and iron influx through iron‐loaded TF

**FIGURE 2:**
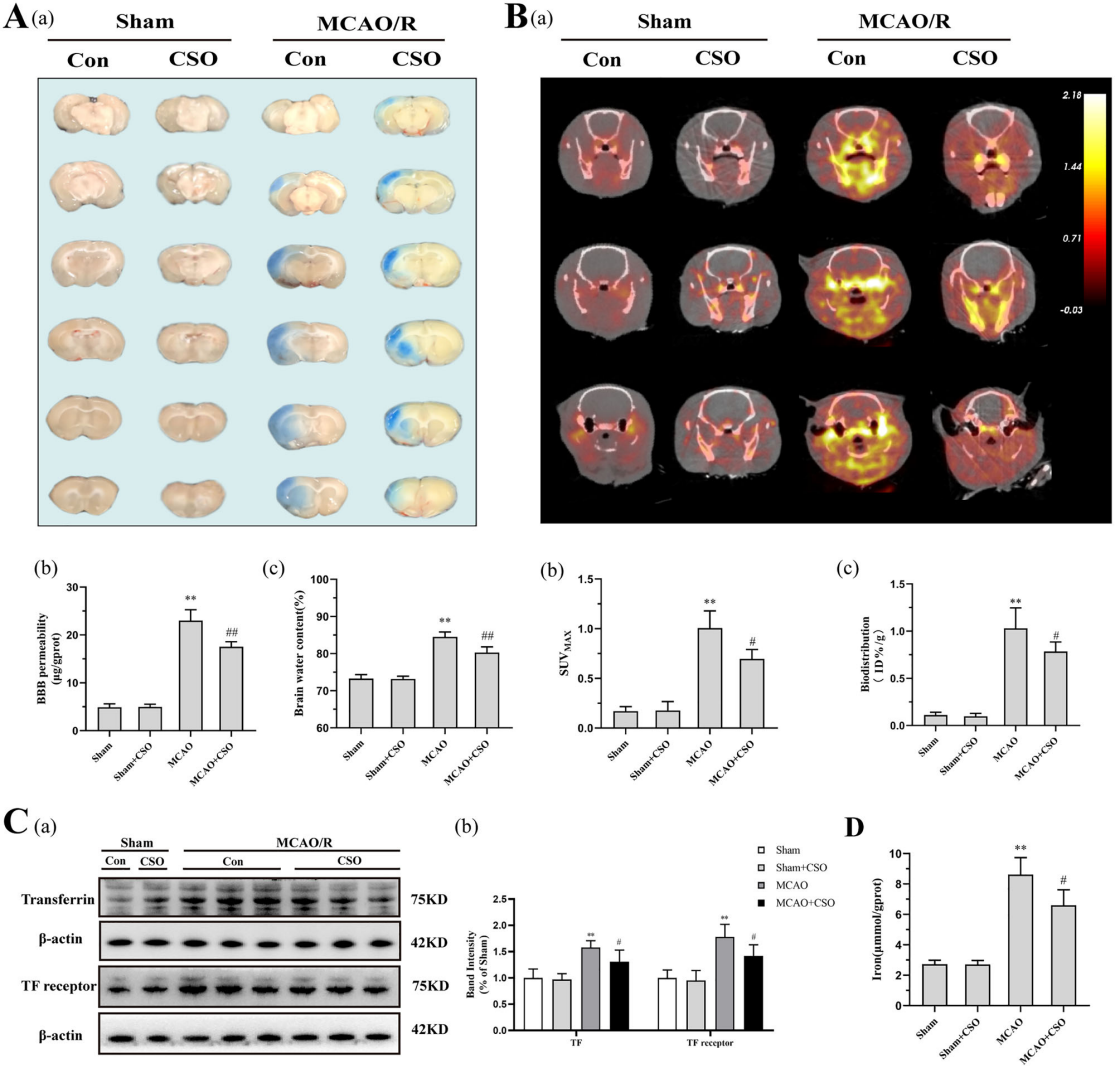
Cottonseed oil (CSO) treatment prevented blood–brain barrier (BBB) damage and iron influx. (A‐a) Representative photographs of brain slices showing the extravasation of Evans blue 24 h after reperfusion. (A‐b) Quantitative analysis of Evans blue leakage in rats from different groups 24 h after reperfusion. **p < .01 versus Sham‐Con group, ^##^ *p* < .01 versus MCAO‐Con group, *n* = 6 per group. (A‐c) Quantification of brain water content in ischemic hemisphere isolated from different groups. **p < .01 versus Sham‐Con group, ^##^ *p* < .01 versus MCAO‐Con group, *n* = 6 per group. (B‐a) Representative PET images of ^68^Ga‐citrate distribution in rat brains from different groups 24 h after reperfusion. (B‐b) SUVmax of ^68^Ga‐citrate in ROI of rats from different groups 24 h after reperfusion. **p < .01 versus Sham‐Con group, ^#^ *p* < .05 versus MCAO‐Con group, *n* = 3 per group. (B‐c) Quantification of ^68^Ga‐citrate biodistribution in ischemic hemisphere of rats from different groups 24 h after reperfusion. **p < .01 versus Sham‐Con group, ^#^ *p* < .05 versus MCAO‐Con group, *n* = 5 per group. (C‐a) Graph of Bands showing the protein expression of the TF and TF receptor in ischemic hemisphere 24 h after reperfusion. (C‐b) Graph showing the expression levels of TF and TF receptor. **p < .01 versus Sham‐Con group, ^#^ *p* < .05 versus MCAO‐Con group, *n* = 6 per group. (D) Levels of iron in ischemic hemisphere from different groups 24 h after reperfusion. **p < .01 versus Sham‐Con group, ^#^ *p* < .05 versus MCAO‐Con group, *n* = 6 per group.

As BBB disruption is the main pathological mechanism of ferroptosis in ischemic stroke, we used EB test and brain water content measurement to determine BBB permeability and edema 24 h after reperfusion. As shown in Figure 2A‐a, the blue staining area indicated the infiltration of Evens blue following BBB destruction; there was no blue color staining in the brain sections of the Sham and Sham+CSO groups, while obvious blue staining areas were presented in the MCAO group. The blue areas in the MCAO+CSO group were smaller than the MCAO group. The EB test showed a significant effect across all four groups (*F*(3,20) = 279.3, *p* = .001). Post hoc comparisons using Tukey HSD test indicated that the EB concentration in the MCAO group (18.7 ± 2.8 μg/g) was significantly higher than the Sham (4.9 ± 0.7 μg/g) and Sham+CSO (5.0 ± 0.6 μg/g) groups. Rats in the MCAO+CSO group (18.7 ± 2.8 μg/g) had decreased EB content than those in the MCAO group. As shown in Figure 2A‐c, there was also a significant effect for brain water content across all four groups (*F*(3,20) = 131.6, *p =* .001). According to the post hoc comparisons using Tukey HSD test, brain water in the MCAO group (83.3 ± 2.7%) was significantly higher than the Sham (73.3 ± 1.1%) and Sham+CSO (73.2 ± 0.7%) groups. Rats in the MCAO+CSO group (80.5 ± 1.9%) had decreased water content than those in the MCAO group.

We used ^68^Ga‐citrate as the probe for PET scan and biodistribution to investigate the level of TF influx and distribution in brain after ischemia–reperfusion injuries. As shown in Figure 2B‐a, there was nearly no highlight region in the Sham and Sham+CSO groups, while larger regions with high ^68^Ga‐Citrate uptake were present in the MCAO group compared to the MCAO+CSO group. A considerable effect for the SUVmax across all four groups is shown in Figure 2B‐b (*F*(3,8) = 41.57, *p* = .001). According to the post hoc comparisons using Tukey HSD test, the SUVmax in the MCAO group (1.0 ± 0.2) was significantly higher than the Sham (0.2 ± 0.1) and Sham+CSO (0.2 ± 0.1) groups. Rats in the MCAO+CSO group (0.7 ± 0.1) had lower SUVmax than those in the MCAO group. The results of biodistribution showed similar trend (Figure 2B‐c), and the radio‐activity values had a marked effect across all four groups (*F*(3,16) = 75.89, *p =* .001). Post hoc comparisons using Tukey HSD test suggested that MCAO group (1.0 ± 0.2 ID%/g) had significantly higher biodistribution of ^68^Ga‐citrate than the Sham (0.1 ± 0.1 ID%/g) and the Sham+CSO (0.1 ± 0.1 ID%/g) groups while CSO treatment dramatically reduced this value to 0.8 ± 0.1 ID%/g. Furthermore, we evaluated the TF expression, TF receptor expression, and iron levels in ischemic penumbra 24 h after reperfusion via Western Blot (Figure 2C) and ELISA (Figure 2D). As shown in Figure 2C(a,b), there was a significant effect for TF expression across all four groups (*F*(3,20) = 17.58, *p =* .001). Post hoc comparisons using Tukey HSD test showed that the expression levels of TF in the MCAO group were significantly higher than the Sham and the Sham+CSO groups. Interestingly, TF expression levels in the MCAO+CSO group was markedly reduced than the MCAO group. There was also a similar trend in expression levels of TF receptor as shown in Figure 2C(a,b) (*F*(3,20) = 22.79, *p =* .001). Expression of TF receptor in the MCAO group was significantly increased than the Sham and the Sham+CSO groups and CSO treatment markedly restored the TF receptor expression.

Finally, we identified the levels of iron in bran tissues (Figure 2D). The results showed a significant effect across all four groups (*F*(3,20) = 53.78, *p =* .001). According to the post hoc comparisons using Tukey HSD test, the iron content was similar in the Sham (2.7 ± 0.3 μmmol/gprot) and the Sham+CSO (2.7 ± 0.3 μmmol/gprot) groups, and ischemic injuries significantly increased the iron levels to 8.5 ± 1.4 μmmol/gprot. CSO treatment reduced iron levels to 6.7 ± 1.3 μmmol/gprot, which was considerably lower than the MCAO group.

### 3.3 CSO treatment regulated the expression levels of ferroptosis‐related proteins and inhibited oxidative stress and lipid peroxidation of ischemia penumbra after stroke

**FIGURE 3:**
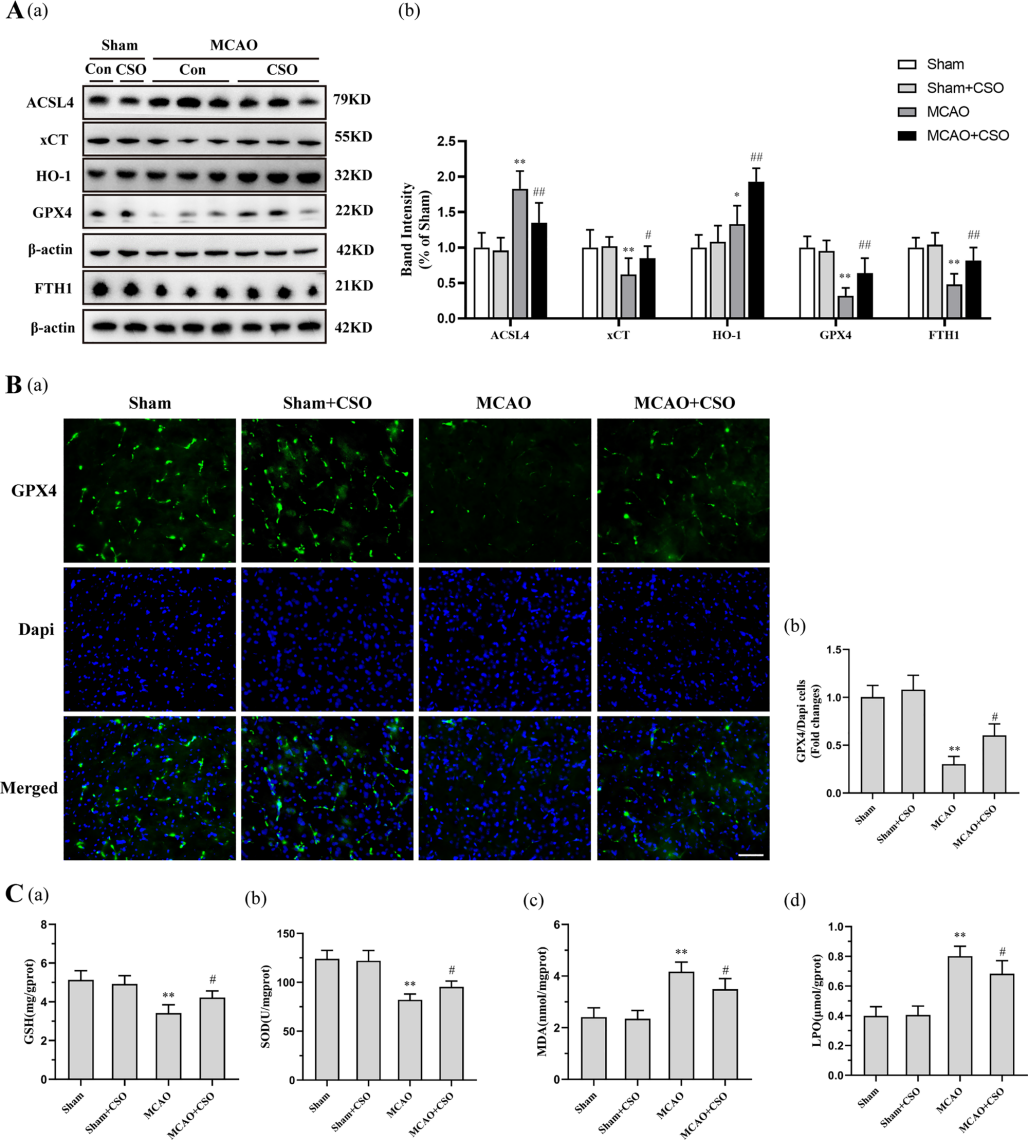
Cottonseed oil (CSO) treatment regulated the ferroptosis related proteins and biomarkers. (A‐a) Representative western blot bands showing protein expression of the ACSL4, xCT, HO1, GPX4, FTH1, β‐actin in ischemic penumbra 24 h after reperfusion. (A‐b) Relative quantification of levels of ACSL4, xCT, HO1, GPX4, FTH1, β‐actin. **p < .01, *p < .05 versus Sham‐Con group, ^##^ *p* < .01, ^#^ *p* < .05 versus MCAO‐Con group, *n* = 6 per group. (B) Immunofluorescence staining of GPX4 in ischemic penumbra 24 h after reperfusion. (a) Representative images showing the GPX4 levels. Scale bars = 20 μm. (b) Relative percentage of GPX4‐positive cells/Dapi‐positive cells according to the immunofluorescence staining. **p < .01 versus Sham‐Con group, ^#^ *p* < .05 versus MCAO‐Con group, *n* = 3 per group. (C) Levels of GSH (a), SOD (b), MDA (c) in ischemic hemisphere from different groups 6 h after reperfusion and LPO levels (d) at 24 h after reperfusion. **p < .01 versus Sham‐Con group, ^##^ *p* < .01, ^#^ *p* < .05 versus MCAO‐Con group, *n* = 6 per group.

The expression levels of ferroptosis‐related proteins 24 h after reperfusion, including GPX4, xCT, FTH1, ACSL4, and HO1, were determined by Western blot. As shown in Figure 3A(a,b), there were significant effects for expression levels of GPX4, xCT, and FTH1 across all four groups (*F*_GPX4_(3,20) = 25.26, *p*_GPX4_= .001; *F*_xCT_(3,20) = 8.073*_,_ p*_xCT_ *=* .003; *F*_FTH1_(3,20) = 15.13, *p*_FTH1_ *=* .001). Post hoc comparisons using Tukey HSD test showed that expression levels of GPX4, xCT, and FTH1 were significantly reduced in MCAO compared to the Sham and Sham+CSO groups, and CSO treatment restored the expression of those proteins in the MCAO+CSO group. There was also a marked effect for expression of ACSL4, which is a key inducer of ferroptosis (*F*(3,20) = 17.96, *p =* .001). Post hoc comparisons using Tukey HSD test indicated that expression levels of ACSL4 were significantly increased in MCAO compared to the Sham and Sham+CSO groups, and CSO treatment significantly reduced the expression of ACSL4 in MCAO+CSO group than those in MCAO group. The quantitative analysis of HO1 expression had an obvious effect across all four groups (*F*(3,20) = 22.49, *p =* .001). Interestingly, HO1 which is an antioxidant stress protein got prominently increased in MCAO group than the Sham and Sham+CSO groups, and CSO treatment further up regulated this protein according to post hoc comparisons using Tukey HSD test.

As shown in Figure 3B(a,b), the immunofluorescence staining was used to detect the expression of GPX4 in ischemic penumbra 24 h after reperfusion. A significant effect for TF expression across all four groups was found in statistical analysis (*F*(3,8) = 36.14, *p =* .001). Post hoc comparisons using Tukey HSD test suggested that expression levels of GPX4 in MCAO group were significantly lower than the Sham and the Sham+CSO groups. However, CSO treatment significantly restored the trend of GPX4 expression levels in MCAO+CSO group.

Oxidative stress and lipid peroxidation make up the critical process of ferroptosis. We measured the levels of GSH, SOD, MDA, and LPO to determine the degree of ferroptosis in the ischemic penumbra (Figure 3C). As shown in Figure 3C‐a, there was a significant effect for the GSH levels across all four groups (*F*(3,20) = 20.46, *p =* .001). Post hoc comparisons using Tukey HSD test suggested that the GSH levels for the MCAO group (3.4 ± 0.4 mg/gprot) were significantly larger than the Sham (5.1 ± 0.5 mg/gprot) and Sham+CSO (4.9 ± 0.4 mg/gprot) groups. Rats in the MCAO+CSO group had higher GSH levels than those in the MCAO group (4.2 ± 0.3 mg/gprot). As shown in Figure 3C‐b, SOD levels were 124.1 ± 8.6, 122.1 ± 10.4, 82.2 ± 5.9, and 95.3 ± 6.0 U/gprot, in the Sham, Sham+CSO, MCAO, and MCAO+CSO groups, respectively. There was also a significant effect for the SOD levels across all four groups (*F*(3,20) = 39.78, *p =* .001). According to post hoc comparisons using Tukey HSD test, SOD levels were significantly decreased in MCAO group compared to the Sham and the Sham+CSO groups, and again CSO treatment counteracted this effect. MDA and LPO are lipid peroxides which can reflect the degree of ferroptosis. As shown in Figure 3C(c,d), the levels of MDA were 2.4 ± 0.4, 2.3 ± 0.3, 4.2 ± 0.4, and 3.5 ± 0.4 nmol/mgprot, in the Sham, Sham+CSO, MCAO, and MCAO+CSO groups, respectively, and the levels of LPS were 0.4 ± 0.1, 0.4 ± 0.1, 0.8 ± 0.1, and 0.7 ± 0.1 μmol/gprot, in the Sham, Sham+CSO, MCAO, and MCAO+CSO groups, respectively; and significant effects for MDA and LPO levels were found across all four groups, (*F*_MDA_(3,20) = 35.47, *p*_MDA_ *=* .001; *F*_LPO_(3,20) = 50.68, *p*_LPO_ *=* .001). Post hoc comparisons using Tukey HSD test suggested that both the levels of MDA and LPO were significantly increased in MCAO group than those in the Sham and Sham+CSO groups, and CSO treatment significantly decreased the levels of MDA and LPO in the MCAO+CSO group compared to the MCAO group, indicating the protective effect of CSO on oxidative stress and lipid peroxidation.

### 3.4 CSO treatment significantly attenuated ferroptosis‐characteristic mitochondrial injury induced by ischemic stroke

**FIGURE 4:**
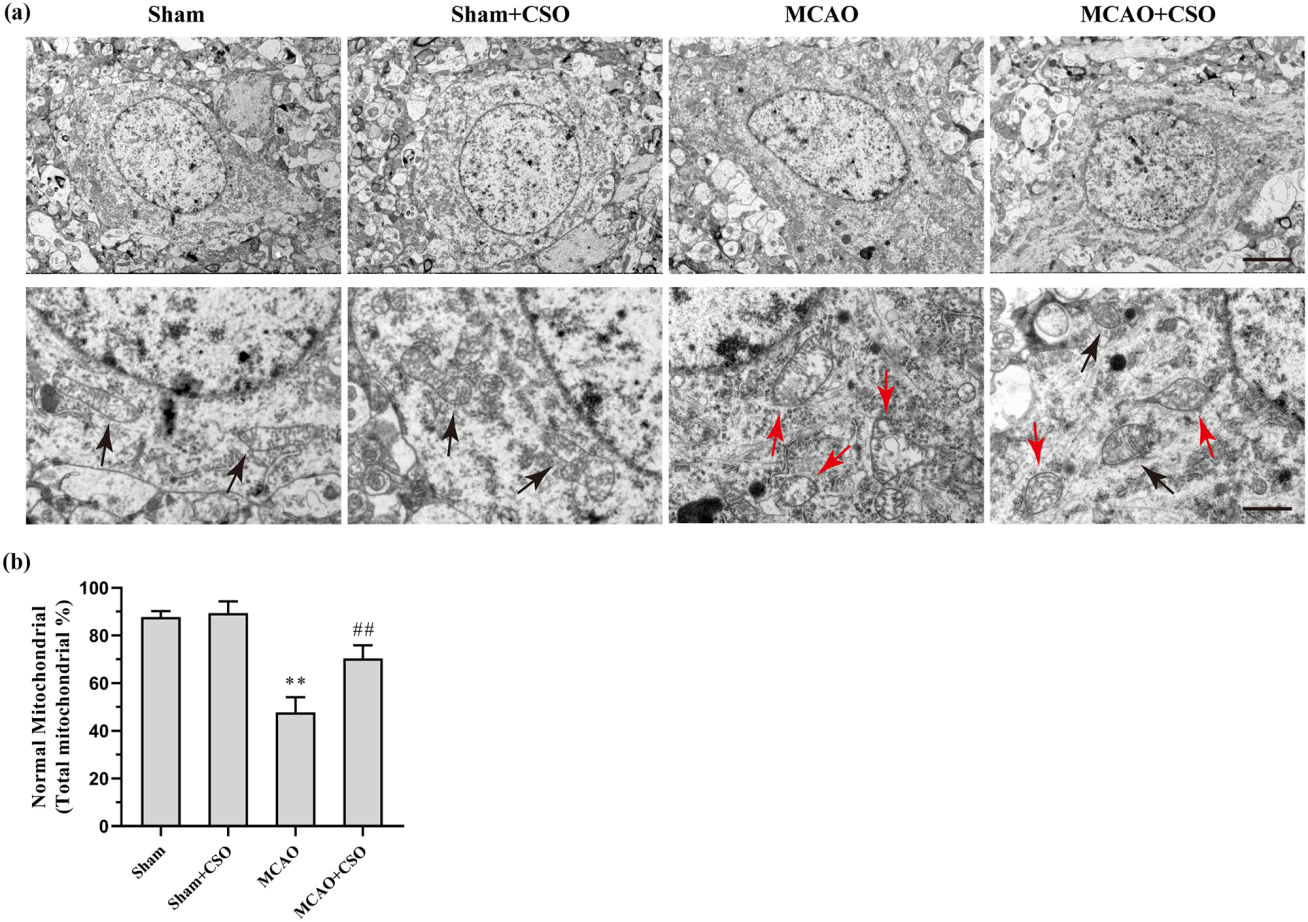
Cottonseed oil (CSO) treatment protect the mitochondrion from ferroptosis injury. (a) Representative photographs of electron microscopy showing the mitochondrial morphology in ischemic penumbra 24 h after reperfusion. Scale bars = 2 μm (top) and 100 nm (bottom). Black arrows indicate normal mitochondrion with contact structure and clear inner ridges. Red arrows represent abnormal mitochondrion with shrinking volume and dense inner ridges. (b) Quantitative analysis of the percentage of normal mitochondrion. **p < .01 versus Sham‐Con group, ^##^ *p* < .01 versus MCAO‐Con group, *n* = 3 per group.

Ferroptosis can induce mitochondrial injuries with specific features, so we used electron microscopy to assess mitochondrial morphology in ischemic penumbra 24 h after reperfusion. As shown in Figure 4a, abnormal mitochondrial morphology was induced by ferroptosis in MCAO group, presented by smaller volume mitochondria compared to the Sham and Sham+CSO group, with condensed mitochondrial membrane densities, reduced or disappeared mitochondria crista, and ruptured outer mitochondrial membrane. The proportions of normal mitochondria without ferroptosis features were 87.7 ± 2.5%, 89.3 ± 5.0%, 47.7 ± 6.4%, 70.3 ± 5.5%, in the Sham, Sham+CSO, MCAO, MCAO+CSO groups, respectively (Figure 4b). The statistical analysis showed a significant effect for the proportion of normal mitochondria across all four groups (*F*(3,8) = 44.11, *p* = .001). Post hoc comparisons using Tukey HSD test indicated that the proportion of normal mitochondria in the MCAO group was significantly lower than those in the Sham and MCAO+CSO groups.

## 4 DISCUSSION

Ischemic heart disease and stroke are the leading causes of death worldwide with increasing prevalence due to overall aging population (Cai et al., 2017). Limited by the narrow treatment window of intravenous rt‐PA, patients seldom get opportune rescue after ischemic stroke (Sandercock et al., 2012). Aside from clinical treatment, prevention of stroke onset appears critical and multiple methods or drugs have tried to reduce the risk or severity of stroke (Phipps & Cronin, 2020).

CSO contains 52%−65% linoleic acid, 25%−36% oleic acid, and 6%−8% palmitic acid (Jones & King, 1996), and is a natural product of plants with high yield and low prices. CSO was reported to attenuate intestinal inflammation via the reduction of inflammatory cytokines, such as TNF‐α, IL‐1β, IL‐6, and IL‐17 (Park et al., 2019). Additionally, CSO could be used as a source of fat to prevent atherosclerosis in dogs (Mahley et al., 1976). Thus, we first explored the effect of CSO on ischemic stroke injury in rats and found that pre‐treatment of CSO could significantly alleviate ischemic stroke injury (Liu et al., 2020). We found the neuroprotective effect of CSO on ischemic stroke injuries and its ability to suppress oxidative stress through nuclear factor erythroid 2‐related factor 2 (Nrf2) pathway (Liu et al., 2021). Moreover, CSO treatment could alleviate neuroinflammation by inhibiting Toll‐like receptor 4 (TLR4)‐nuclear factor kappa B (NF‐κB) pathway and the over‐activation of microglia and astrocyte (Liu et al., 2020). In this study, the protective effect of CSO on ischemic stroke injury was further proofed. As an edible oil, CSO should be taken into consideration a new preventive strategy of neural injuries after stroke.

Ferroptosis is characterized by lipid peroxidation mediated by ferrous iron, involved in the neural damage during stroke and other central nervous diseases (Shen et al., 2020). As the pathological process of ferroptosis involves oxidative stress and BBB disruption during ischemic stroke, we tried to explore the effect of CSO on ferroptosis to inhibit oxidative stress and neuroinflammation. Iron is the most abundant metal in brain and plays an important role in normal neural functions, but abnormal accumulation of iron has been found in numerous neural diseases (Qian & Ke, 2019). The modulation of BBB to balance iron transport by iron‐loaded TF is regarded as the main uptake pathway in brain (Qian & Ke, 2019).

Upon ischemic stroke, the BBB was disrupted and led to increased iron influx through iron‐loaded TF. This mechanism starts with TF interaction with TF receptor, and iron is transported into cells and delivered to the labile iron pool (LIP) by divalent metal iron transporter 1 (DMT1). The ferrous ion is then released from ferritin via autophagic degradation mediated by nuclear receptor coactivator 4 (NCOA4) leading to lipid peroxides resulting from Fenton reaction (Zhou et al., 2021). Iron is essential to cause ferroptosis, and the administration of inhibitors of ferroptosis or iron chelators can effectively alleviate neural injuries after stroke (Chen et al., 2021; Zhu et al., 2022). Moreover, intracellular iron overload can be prevented by the iron‐storage protein ferritin. Knockdown of ferritin heavy chain (FTH1) can aggravate ferroptosis while promoting FTH1 expression can restrain ferroptosis and ferritinophagy (Tian et al., 2020). We found that ischemia reperfusion injury decreased FTH1 expression while CSO markedly reversed that trend. TF expression and iron levels decrease upon CSO treatment, as shown by Western Blot and ELISA, but we also confirmed iron level biodistribution with ^68^Ga‐citrate, which can simulate free iron binding to TF in circulation and measured radiation technology (Vorster et al., 2018). ^68^Ga‐citrate has been generally applied in infectious diseases and tumors (Behr et al., 2016; Nanni et al., 2010), but this is the first study to apply ^68^Ga‐citrate in MCAO‐R model. Compared with common study methods, PET can reflect the pathological condition in living animals allowing for a more accurate measurement. Regardless of the endpoint, CSO protected BBB function caused by ischemic injuries (EB test and water content) and prevented iron‐loaded TF influx to the lesion sites due to the lower BBB permeability.

To date, there is no gold standard to determine ferroptosis. GPX4 has been reported as a key protein during ferroptosis due to its effect on lipid peroxides (Dixon et al., 2012). Depletion of GPX4 was observed during stroke‐induced ferroptosis, and up‐regulation of GPX4 expression can protect neural functions from ferroptosis injuries (Alim et al., 2019). Another specific feature of ferroptosis is the exhaustion of antioxidants such as GSH and the accumulation of lipid peroxides like MDA, LPO, and 4‐hydroxynonenal (4‐HNE) (Park et al., 2021). The cystine glutamate antiporter xCT plays an important role in ferroptosis, it can transport the cystine into cells for GSH synthesis, and the ferroptosis inducer erastin can inhibit its function, synergizing with GPX 4 and GSH to form a catalytic cycle to act as antioxidants. Blockage of xCT‐GSH‐GPX4 axis can worsen ferroptosis (Alim et al., 2019). In our study, CSO up‐regulated the expression level of xCT and GPX4, increased antioxidant activity including GSH, SOD, and GSH‐PX, and decreased levels of MDA and LPO. Our previous study showed CSO can alleviate oxidative stress by activating Nrf2, which was reported to reduce ferroptosis and up‐regulate the expression of xCT and HO1 (Dong et al., 2020). Therefore, we measured the expression levels of HO1 by western blot. Consistent with previous observations, HO1 levels were elevated after MCAO‐R injury compared with the sham group, but further significantly up‐regulated during CSO treatment. Hence, we speculate that the increased HO1 expression induced by ischemic stroke may be a compensatory change in self‐protection and CSO treatment could further up‐regulat antioxidative stress‐related proteins and molecules to alleviate lipid peroxidation.

Another key regulator of ferroptosis is ACSL4. ACSL4 is an important isozyme for polyunsaturated fatty acid (PUFA) metabolism that catalyzes the synthesis of long‐chain polyunsaturated CoAs with a preference for arachidonic acid. ACSL4 induces PUFA esterification into phospholipids, thus promoting neuronal death by ferroptosis during stroke injuries (Chen et al., 2021). GPX4‐ACSL4 double‐knockout cells are resistant to ferroptosis (Doll et al., 2017), indicating the essential role of ACSL4 in ferroptosis. In this study, CSO treatment significantly decreased the expression level of ACSL4 and inhibition of ACSL4 reduced lipids prone to peroxidation alleviating ferroptosis. Nevertheless, the mechanism of CSO treatment to modulate lipid metabolism needs further research.

Mitochondria are the central sites for oxygen consumption to produce ATP and maintain normal physical function. Along with the oxidative metabolism, ROS are also generated in large quantities (Shadel & Horvath, 2015), which induce ferroptosis in the membranous lipid of mitochondria. Mitochondrial morphological abnormalities and dysfunction are typical characteristics of ferroptosis. The mitochondrion becomes atrophied and the mitochondrial inner membrane ridge disappears with intact outer membrane (Li et al., 2021). Besides the GPX4 antioxidant system, there is another mitochondria‐independent ferroptosis defense enzyme: the dihydroorotate dehydrogenase which can reduce the ubiquinone (CoQ) to ubiquinol (CoQH_2_) to decrease lipid peroxides (Mao et al., 2021). In our study, CSO treatment improved mitochondrial morphology by reducing ischemia‐related ferroptosis and oxidative stress. However, the influence of CSO treatment on mitochondria‐specific anti‐ferroptosis system should be further explored in future.

There were still some limitations in the present study. First, the resolution ratio of CT scan could not analyze the exact location of the PET images. Limited by the research capabilities, we could not combine PET with MRI or enhanced CT. Second, the exact signaling mechanisms or pathways following CSO treatment and their crosstalk with ferroptosis after ischemic stroke remain largely unknown and need to be further explored.

## 5 CONCLUSION

In conclusion, the current study revealed that CSO can alleviate ischemic stroke injuries by protecting from ferroptosis and the underlying mechanism may be related to the reduction of iron and lipid peroxide levels, enhancement of anti‐oxidative stress through xCT‐GSH‐GPX4 axis and inhibition of ACSL4 expression. Combined with our previous studies, we suggest that CSO may be a potential preventive strategy for ischemic stroke injury.

## AUTHOR CONTRIBUTIONS

Miao Sun and Min Liu conducted most of the experiments and drafted the manuscript. Qingxiao Li contributed to the nuclear medicine experiments. Xiaoying Zhang and Siyuan Liu contributed to statistical analysis and manuscript editing. Huikai Yang and Le Yang contributed to data interruption. Yulong Ma, Weidong Mi, and Jiahe Tian designed this study and edited the manuscript. All authors read and approved the final manuscript.

## CONFLICT OF INTEREST STATEMENT

The authors declare no conflicts of interest.

### PEER REVIEW

The peer review history for this article is available at https://publons.com/publon/10.1002/brb3.3179

## Supporting information

Supp Information

## Data Availability

The datasets during and/or analyzed during the current study are available from the corresponding author on reasonable request.

## References

[brb33179-bib-0001] Aizawa, N. , Iijima, K. , Rosenbaum, J. S. , Downs, T. R. , Igawa, Y. , Andersson, K.‐E. , & Wyndaele, J.‐J. (2011). Comparison of the effects of oestrogen deficiency and old age on primary bladder afferent activity and voiding behaviour in the ageing female rat. Bju International, 108(2 Pt 2), E10–E16. 10.1111/j.1464-410X.2010.09689.x 20875090

[brb33179-bib-0002] Alim, I. , Caulfield, J. T. , Chen, Y. , Swarup, V. , Geschwind, D. H. , Ivanova, E. , Seravalli, J. , Ai, Y. , Sansing, L. H. , Ste Marie, E. J. , Hondal, R. J. , Mukherjee, S. , Cave, J. W. , Sagdullaev, B. T. , Karuppagounder, S. S. , & Ratan, R. R. (2019). Selenium drives a transcriptional adaptive program to block ferroptosis and treat stroke. Cell, 177(5), 1262–1279.e25. 10.1016/j.cell.2019.03.032 31056284

[brb33179-bib-0003] Araujo, R. S. , Oliveira, A. C. , Sousa, F. C. B. , Dourado, L. R. B. , Guimarães, S. E. F. , Silva, W. , Biagiotti, D. , Bayão, G. F. V. , & Sousa, K. R. S. (2019). Effects of cottonseed oil and ferrous sulfate on the performance and expression of antioxidant enzymes in broilers. Poultry Science, 98(9), 3860–3869. 10.3382/ps/pez103 30877746

[brb33179-bib-0004] Bachour, S. P. , Hevesi, M. , Bachour, O. , Sweis, B. M. , Mahmoudi, J. , Brekke, J. A. , & Divani, A. A. (2016). Comparisons between Garcia, Modo, and Longa rodent stroke scales: Optimizing resource allocation in rat models of focal middle cerebral artery occlusion. Journal of the Neurological Sciences, 364, 136–140. 10.1016/j.jns.2016.03.029 27084232

[brb33179-bib-0005] Behr, S. C. , Aggarwal, R. , Seo, Y. , Aparici, C. M. , Chang, E. , Gao, K. T. , Tao, D. H. , Small, E. J. , & Evans, M. J. (2016). A feasibility study showing [68Ga]citrate PET detects prostate cancer. Molecular Imaging and Biology, 18(6), 946–951. 10.1007/s11307-016-0966-5 27184068 PMC6430569

[brb33179-bib-0006] Behr, S. C. , Villanueva‐Meyer, J. E. , Li, Y. , Wang, Y.‐H. , Wei, J. , Moroz, A. , Lee, J. K. L. , Hsiao, J. C. , Gao, K. T. , Ma, W. , Cha, S. , Wilson, D. M. , Seo, Y. , Nelson, S. J. , Chang, S. M. , & Evans, M. J. (2018). Targeting iron metabolism in high‐grade glioma with 68Ga‐citrate PET/MR. JCI Insight, 2, 3(21), e93999. 10.1172/jci.insight.93999 30385712 PMC6238742

[brb33179-bib-0007] Cai, W. , Zhang, K. , Li, P. , Zhu, L. , Xu, J. , Yang, B. , Hu, X. , Lu, Z. , & Chen, J. (2017). Dysfunction of the neurovascular unit in ischemic stroke and neurodegenerative diseases: An aging effect. Ageing Research Reviews, 34, 77–87. 10.1016/j.arr.2016.09.006 27697546 PMC5384332

[brb33179-bib-0008] Campbell, B. C. V. , De Silva, D. A. , Macleod, M. R. , Coutts, S. B. , Schwamm, L. H. , Davis, S. M. , & Donnan, G. A. (2019). Ischaemic stroke. Nature reviews Disease primers, 10, 5(1), 70. 10.1038/s41572-019-0118-8 31601801

[brb33179-bib-0009] Chen, J. , Yang, L. , Geng, L. , He, J. , Chen, L. , Sun, Q. , Zhao, J. , & Wang, X. (2021). Inhibition of acyl‐CoA synthetase long‐chain family member 4 facilitates neurological recovery after stroke by regulation ferroptosis. Front Cell Neurosci, 15, 632354. 10.3389/fncel.2021.632354 33889074 PMC8055945

[brb33179-bib-0010] Dixon, S. J. , Lemberg, K. M. , Lamprecht, M. R. , Skouta, R. , Zaitsev, E. M. , Gleason, C. E. , Patel, D. N. , Bauer, A. J. , Cantley, A. M. , Yang, W. S. , Morrison, B. , & Stockwell, B. R. (2012). Ferroptosis: An iron‐dependent form of nonapoptotic cell death. Cell, 149, 1060–1072. 10.1016/j.cell.2012.03.042 22632970 PMC3367386

[brb33179-bib-0011] Doll, S. , Proneth, B. , Tyurina, Y. Y. , Panzilius, E. , Kobayashi, S. , Ingold, I. , Irmler, M. , Beckers, J. , Aichler, M. , Walch, A. , Prokisch, H. , Trümbach, D. , Mao, G. , Qu, F. , Bayir, H. , Füllekrug, J. , Scheel, C. H. , Wurst, W. , Schick, J. A. , … Conrad, M. (2017). ACSL4 dictates ferroptosis sensitivity by shaping cellular lipid composition. Nature Chemical Biology, 13(1), 91–98. 10.1038/nchembio.2239 27842070 PMC5610546

[brb33179-bib-0012] Dong, H. , Qiang, Z. , Chai, D. , Peng, J. , Xia, Y. , Hu, R. , & Jiang, H. (2020). Nrf2 inhibits ferroptosis and protects against acute lung injury due to intestinal ischemia reperfusion via regulating SLC7A11 and HO‐1. Aging (Albany NY), 12(13), 12943–12959. 10.18632/aging.103378 32601262 PMC7377827

[brb33179-bib-0013] Jones, L. A. , & King, C. C. (1996). Edible oils and fat products: Oils and oil seeds (pp. 159–240). John Wiley.

[brb33179-bib-0014] Li, G. , Li, X. , Dong, J. , & Han, Y. (2021). Electroacupuncture ameliorates cerebral ischemic injury by inhibiting ferroptosis. Front Neurol, 12, 619043. 10.3389/fneur.2021.619043 33763013 PMC7982901

[brb33179-bib-0015] Liu, M. , Li, H. , Zhang, L. , Xu, Z. , Song, Y. , Wang, X. , Chu, R. , Xiao, Y. , Sun, M. , Ma, Y. , & Mi, W. (2021). Cottonseed oil alleviates ischemic stroke‐induced oxidative stress injury via activating the Nrf2 signaling pathway. Molecular Neurobiology, 58(6), 2494–2507. 10.1007/s12035-020-02256-y 33443681

[brb33179-bib-0016] Liu, M. , Xu, Z. , Wang, L. , Zhang, L. , Liu, Y. , Cao, J. , Fu, Q. , Liu, Y. , Li, H. , Lou, J. , Hou, W. , Mi, W. , & Ma, Y. (2020). Cottonseed oil alleviates ischemic stroke injury by inhibiting the inflammatory activation of microglia and astrocyte. J Neuroinflammation, 17(1), 270. 10.1186/s12974-020-01946-7 32917229 PMC7488511

[brb33179-bib-0017] Mahley, R. W. , Nelson, A. W. , Ferrans, V. J. , & Fry, D. L. (1976). Thrombosis in association with atherosclerosis induced by dietary perturbations in dogs. Science, 192(4244), 1139–1141. 10.1126/science.1273587 1273587

[brb33179-bib-0018] Mao, C. , Liu, X. , Zhang, Y. , Lei, G. , Yan, Y. , Lee, H. , Koppula, P. , Wu, S. , Zhuang, L. , Fang, B. , Poyurovsky, M. V. , Olszewski, K. , & Gan, B. (2021). DHODH‐mediated ferroptosis defence is a targetable vulnerability in cancer. Nature, 593(7860), 586–590. 10.1038/s41586-021-03539-7 33981038 PMC8895686

[brb33179-bib-0019] Miura, D. , Kida, Y. , & Nojima, H. (2007). Camellia oil and its distillate fractions effectively inhibit the spontaneous metastasis of mouse melanoma BL6 cells. FEBS Letters, 581(13), 2541–2548. 10.1016/j.febslet.2007.04.080 17499720

[brb33179-bib-0020] Nanni, C. , Errani, C. , Boriani, L. , Fantini, L. , Ambrosini, V. , Boschi, S. , Rubello, D. , Pettinato, C. , Mercuri, M. , Gasbarrini, A. , & Fanti, S. (2010). 68Ga‐citrate PET/CT for evaluating patients with infections of the bone: Preliminary results. Journal of Nuclear Medicine, 51(12), 1932–1936. 10.2967/jnumed.110.080184 21078801

[brb33179-bib-0021] Park, J.‐S. , Choi, J. , Hwang, S.‐H. , Kim, J.‐K. , Kim, E.‐K. , Lee, S.‐Y. , Lee, B.‐I. , Park, S.‐H. , & Cho, M.‐L. (2019). Cottonseed Oil Protects Against Intestinal Inflammation in Dextran Sodium Sulfate‐Induced Inflammatory Bowel Disease. Journal of Medicinal Food, 22(7), 672–679. 10.1089/jmf.2018.4323 31112045

[brb33179-bib-0022] Park, M. W. , Cha, H. W. , Kim, J. , Kim, J. H. , Yang, H. , Yoon, S. , Boonpraman, N. , Yi, S. S. , Yoo, I. D. , & Moon, J.‐S. (2021). NOX4 promotes ferroptosis of astrocytes by oxidative stress‐induced lipid peroxidation via the impairment of mitochondrial metabolism in Alzheimer's diseases. Redox Biology, 41, 101947. 10.1016/j.redox.2021.101947 33774476 PMC8027773

[brb33179-bib-0023] Phipps, M. S. , & Cronin, C. A. (2020). Management of acute ischemic stroke. Bmj, 368, l6983. 10.1136/bmj.l6983 32054610

[brb33179-bib-0024] Qian, Z.‐M. , & Ke, Y. (2019). Brain iron transport. Biological Reviews of the Cambridge Philosophical Society, 94(5), 1672–1684. 10.1111/brv.12521 31190441

[brb33179-bib-0025] IST‐3 collaborative group , Sandercock, P. , Wardlaw, J. M. , Lindley, R. I. , Dennis, M. , Cohen, G. , Murray, G. , Innes, K. , Venables, G. , Czlonkowska, A. , Kobayashi, A. , Ricci, S. , Murray, V. , Berge, E. , Slot, K. B. , Hankey, G. J. , Correia, M. , Peeters, A. , Matz, K. , Lyrer, P. , … Arauz, A. . (2012). The benefits and harms of intravenous thrombolysis with recombinant tissue plasminogen activator within 6 h of acute ischaemic stroke (the third international stroke trial [IST‐3]): A randomised controlled trial. Lancet, 379(9834), 2352–2363. 10.1016/S0140-6736(12)60768-5 22632908 PMC3386495

[brb33179-bib-0026] Shadel, G. S. , & Horvath, T. L. (2015). Mitochondrial ROS signaling in organismal homeostasis. Cell, 163(3), 560–569. 10.1016/j.cell.2015.10.001 26496603 PMC4634671

[brb33179-bib-0027] Shen, L. , Lin, D. , Li, X. , Wu, H. , Lenahan, C. , Pan, Y. , Xu, W. , Chen, Y. , Shao, A. , & Zhang, J. (2020). Ferroptosis in acute central nervous system injuries: The future direction? Frontiers in Cell and Developmental Biology, 8, 594. 10.3389/fcell.2020.00594 32760721 PMC7373735

[brb33179-bib-0028] Tian, Y. , Lu, J. , Hao, X. , Li, H. , Zhang, G. , Liu, X. , Li, X. , Zhao, C. , Kuang, W. , Chen, D. , & Zhu, M. (2020). FTH1 inhibits ferroptosis through ferritinophagy in the 6‐OHDA model of Parkinson's disease. Neurotherapeutics, 17(4), 1796–1812. 10.1007/s13311-020-00929-z 32959272 PMC7851296

[brb33179-bib-0029] Vorster, M. , Buscombe, J. , Saad, Z. , & Sathekge, M. (2018). Past and future of Ga‐citrate for infection and inflammation imaging. Current Pharmaceutical Design, 24(7), 787–794. 10.2174/1381612824666171129200611 29189131

[brb33179-bib-0030] Yamasaki, T. , Hatori, A. , Zhang, Y. , Mori, W. , Kurihara, Y. , Ogawa, M. , Wakizaka, H. , Rong, J. , Wang, L. , Liang, S. , & Zhang, M.‐R. (2021). Neuroprotective effects of minocycline and KML29, a potent inhibitor of monoacylglycerol lipase, in an experimental stroke model: A small‐animal positron emission tomography study. Theranostics, 11(19), 9492–9502. 10.7150/thno.64320 34646382 PMC8490517

[brb33179-bib-0031] Yeo, L. L. L. , Paliwal, P. , Teoh, H. L. , Seet, R. C. , Chan, B. P. L. , Liang, S. , Venketasubramanian, N. , Rathakrishnan, R. , Ahmad, A. , Ng, K. W. P. , Loh, P. K. , Ong, J. J. Y. , Wakerley, B. R. , Chong, V. F. , Bathla, G. , & Sharma, V. K. (2013). Timing of recanalization after intravenous thrombolysis and functional outcomes after acute ischemic stroke. JAMA Neurology, 70(3), 353–358. 10.1001/2013.jamaneurol.547 23599933

[brb33179-bib-0032] Zhou, Y. , Liao, J. , Mei, Z. , Liu, X. , & Ge, J. (2021). Insight into crosstalk between ferroptosis and necroptosis: Novel therapeutics in ischemic stroke. Oxid Med Cell Longev, 2021, 9991001. 10.1155/2021/9991001 34257829 PMC8257382

[brb33179-bib-0033] Zhu, H. , Huang, J. , Chen, Y. , Li, X. , Wen, J. , Tian, M. , Ren, J. , Zhou, L. , & Yang, Q. (2022). Resveratrol pretreatment protects neurons from oxygen‐glucose deprivation/reoxygenation and ischemic injury through inhibiting ferroptosis. Bioscience, Biotechnology, and Biochemistry, 86(6), 704–716. 10.1093/bbb/zbac048 35357412

