# Supplementary material for: Cottonseed oil alleviates ischemic stroke injury by inhibiting ferroptosis
Source: Brain Behav. 2023 Jul 21;13(10):e3179. doi: 10.1002/brb3.3179 (PMC10570467; doi:10.1002/brb3.3179)
Supplement: Supplementary file 1 — Supp Information [file BRB3-13-e3179-s001.docx]

**Supplementary data 1**

| Group | | MABP  (mmHg) | Temp  (oC) | Glu  （dl/ml） | Hct  (%) | pH | pO_2_  (mmHg) | pCO2  (mmHg) |
| --- | --- | --- | --- | --- | --- | --- | --- | --- |
| Sham | pre | 67±1 | 37±0.1 | 188±13 | 31±2 | 7.4±0.1 | 140±2 | 43±3 |
|  | during | 68±2 | 37±0.2 | 191±12 | 30±3 | 7.4±0.1 | 138±3 | 48±5 |
|  | post | 68±1 | 37±0.2 | 171±10 | 29±2 | 7.4±0.1 | 140±3 | 44±5 |
| Sham+CSO | pre | 68±1 | 37±0.1 | 191±13 | 30±1 | 7.4±0.1 | 138±3 | 45±3 |
|  | during | 68±2 | 37±0.2 | 187±14 | 30±2 | 7.4±0.1 | 135±4 | 51±4 |
|  | post | 68±1 | 37±0.2 | 173±11 | 30±2 | 7.4±0.1 | 139±6 | 46±5 |
| MCAO | pre | 67±2 | 37±0.1 | 190±14 | 31±2 | 7.4±0.1 | 142±3 | 47±3 |
|  | during | 68±2 | 37±0.2 | 165±15 | 30±2 | 7.4±0.1 | 137±5 | 51±6 |
|  | post | 65±2 | 37±0.2 | 163±15 | 28±3 | 7.4±0.1 | 135±6 | 45±5 |
| MCAO+CSO | pre | 66±2 | 37±0.1 | 189±14 | 31±3 | 7.4±0.1 | 141±4 | 45±4 |
|  | during | 67±2 | 37±0.2 | 171±13 | 29±3 | 7.4±0.1 | 136±4 | 52±5 |
|  | post | 65±2 | 37±0.2 | 169±14 | 28±3 | 7.4±0.1 | 138±5 | 45±6 |

MABP: mean arterial blood pressure; Temp:rectal temperature; Glu: Glucose; Hct:Hematocrit.
